# Effect of *AMY1* copy number variation and various doses of starch intake on glucose homeostasis: data from a cross-sectional observational study and a crossover meal study

**DOI:** 10.1186/s12263-021-00701-8

**Published:** 2021-11-17

**Authors:** Mary Farrell, Stina Ramne, Phébée Gouinguenet, Louise Brunkwall, Ulrika Ericson, Anne Raben, Peter M. Nilsson, Marju Orho-Melander, Yvonne Granfeldt, Juscelino Tovar, Emily Sonestedt

**Affiliations:** 1 Department of Clinical Sciences Malmö, Lund University, Jan Waldenströms gata 35, CRC 60:13, 214 28 Malmö, Sweden; 2 Department of Food Technology, Engineering and Nutrition, Lund University, Lund, Sweden; 3 Department of Nutrition, Exercise and Sports, University of Copenhagen, Copenhagen, Denmark

**Keywords:** Nutrigenetics, Diet-gene interaction, Salivary α-amylase, *AMY1*, Copy number variation, Starch intake, Metabolic health, Body mass index, Gut microbiome

## Abstract

**Background:**

Copy number (CN) variation (CNV) of the salivary amylase gene (*AMY1*) influences the ability to digest starch and may influence glucose homeostasis, obesity and gut microbiota composition. Hence, the aim was to examine the association of *AMY1* CNV with fasting glucose, BMI, and gut microbiota composition considering habitual starch intake and to investigate the effect of *AMY1* CNV on the postprandial response after two different starch doses.

**Methods:**

The Malmö Offspring Study (*n* = 1764, 18–71 years) was used to assess interaction effects between *AMY1* CNV (genotyped by digital droplet polymerase chain reaction) and starch intake (assessed by 4-day food records) on fasting glucose, BMI, and 64 gut bacteria (16S rRNA sequencing). Participants with low (≤ 4 copies, *n* = 9) and high (≥ 10 copies, *n* = 10) *AMY1* CN were recruited for a crossover meal study to compare postprandial glycemic and insulinemic responses to 40 g and 80 g starch from white wheat bread.

**Results:**

In the observational study, no overall associations were found between *AMY1* CNV and fasting glucose, BMI, or gut microbiota composition. However, interaction effects between *AMY1* CNV and habitual starch intake on fasting glucose (*P* = 0.03) and BMI (*P* = 0.05) were observed, suggesting inverse associations between *AMY1* CNV and fasting glucose and BMI at high starch intake levels and positive association at low starch intake levels. No associations with the gut microbiota were observed. In the meal study, increased postprandial glucose (*P* = 0.02) and insulin (*P* = 0.05) were observed in those with high *AMY1* CN after consuming 40 g starch. This difference was smaller and nonsignificant after consuming 80 g starch.

**Conclusions:**

Starch intake modified the observed association between *AMY1* CNV and fasting glucose and BMI. Furthermore, depending on the starch dose, a higher postprandial glucose and insulin response was observed in individuals with high *AMY1* CN than in those with low *AMY1* CN.

**Trial registration:**

ClinicalTrials.gov, NCT03974126. Registered 4 June 2019—retrospectively registered.

**Supplementary Information:**

The online version contains supplementary material available at 10.1186/s12263-021-00701-8.

## Background

Salivary α-amylase is a digestive enzyme present in the oral cavity. Along with pancreatic amylase, it is responsible for the degradation of starch into maltose, isomaltose, and maltotriose, which are further broken down to glucose in the small intestine by brush border disaccharidases [1]. The gene encoding salivary α-amylase, *AMY1*, exhibits more variation in copy number (CN) than the gene encoding pancreatic amylase, *AMY2*, ranging from 2 to 17 copies, and 0 to 6 copies, respectively [2]. Copy number variation (CNV) occurs when the number of copies of a particular gene differs in comparison to a reference genome, due to either deletion or duplication of specific DNA regions [3]. Humans have much greater variation in CNs of the *AMY1* gene than primates, which have only 0 to 2 copies [4]. Notably, CNV of the *AMY1* gene may be an evolutionary response to the increased amount of starch in the diet following the agricultural revolution. It has been shown that populations with diets historically high in starch have, on average, more copies of this gene than populations with diets lower in starch [4].

Epidemiological research has shown an inverse association between *AMY1* CNV and obesity traits [5–14]; however, findings have been inconclusive or contradictory [15–22]. Only a few studies have examined the association between *AMY1* CNV and fasting glucose homeostasis traits [9, 11, 12, 16, 18, 21, 23] or gut microbiota composition [11, 21, 24]. We have previously observed significant interaction effects between starch intake and *AMY1* CN on BMI [25] and fasting insulin and HOMA-IR [23] in the Malmö Diet and Cancer study, indicating that by taking starch intake into account, we may clarify the relationships between *AMY1* CN and obesity and glucose homeostasis traits. In addition, people with lower *AMY1* CN may have more undigested starch passing through to the large intestine, providing increased substrate for bacterial fermentation, which could impact the gut microbiota composition [24].

Regarding the effect of *AMY1* CNV on postprandial glucose following consumption of starch, very few studies have been carried out, and the findings have been contradictory. A study by Atkinson et al. reported that individuals with high *AMY1* CN showed higher glycemic responses to a high-starch meal [18], whereas Higuchi et al. found no difference in glycemic response between individuals with low and high *AMY1* CN [26]. Other studies have found that those with high salivary α-amylase activity, which is highly correlated with *AMY1* CN [4], had lower glycemia in response to high-starch meals [27, 28]. None of these studies has investigated the effect of *AMY1* CN on postprandial responses to different doses of starch.

The aim was first to examine the cross-sectional associations of *AMY1* CN with fasting glucose, BMI, and gut microbiota composition and the interaction between *AMY1* CN and habitual starch intake in a Swedish population (study 1), and second to investigate the effect of *AMY1* CN on postprandial glucose and insulin response after two different doses of starch in a meal study (study 2).

## Methods

### Study 1—Cross-sectional cohort analysis of associations of *AMY1* CNV with fasting plasma glucose and BMI

#### Study population and design

The Malmö Offspring Study (MOS) is a cohort from southern Sweden initiated in 2013, in which children and grandchildren (age > 18 years) of participants from an earlier cohort, the Malmö Diet and Cancer-Cardiovascular Cohort (MDC-CC), were recruited. Participants were recruited using official register information from the Swedish Tax Agency. Anthropometrics (including weight, height and waist circumference) were measured, and venous blood samples were drawn after an overnight fast. Plasma glucose was measured using a HemoCue Glucose 201+ Analyzer (HemoCue AB, Ängelholm, Sweden). Participants were instructed how to conduct a validated web-based 4-day food record to report their dietary intake [29]. A lifestyle questionnaire was also distributed via email including questions about smoking, alcohol and physical activity habits, socioeconomic factors, family and disease history, etc. Informed written consent was provided by all participants, and ethical permission for MOS protocols was obtained from the regional ethics committee in Lund (Dnr. 2012/594) [30]. Of the first 1869 MOS participants who had DNA extracted, we included 1822 participants for whom *AMY1* CNV was successfully genotyped and further excluded 58 participants with prevalent diabetes identified using disease registers or self-reported in the lifestyle questionnaire, resulting in a total study sample of 1764 participants (Supplementary Figure 1, Additional File 1).

#### Genotyping

Whole blood (4 ml) was taken at the research center by trained nurses using a Venflon catheter and collected into tubes containing ethylenediaminetetraacetic acid. DNA was then extracted from the buffy coat (900 μl) and stored at − 80 °C until DNA analysis. Genotyping of *AMY1* CNV was performed at TATAA Biocenter (Gothenburg, Sweden) in October 2019. The CN for the *AMY1* gene compared to the reference gene *AP3B1* was determined using droplet digital polymerase chain reaction (ddPCR) with the use of a QX200 AutoDG Droplet Digital PCR System (Bio-Rad Laboratories, Hercules, CA). Segments of DNA were amplified using a T100 Thermal Cycler (Bio-Rad Laboratories) for PCR amplification. Samples were analyzed along with a negative control (RNase free water) on each plate. Samples with high CN were diluted and reanalyzed to exclude the possibility of the presence of too much DNA or a technical error. Following amplification, the samples were stored overnight at 4 °C before being read on a Bio-Rad QX200 Droplet Reader. QuantaSoft software version 1.7.4 (Bio-Rad Laboratories) was used to determine the CN. For a subsample of the cohort (*n* = 436), *AMY1* CNV had been genotyped in 2016. The correlation between the two genotyping results was very high (*r* = 0.99).

#### Dietary data

Participants (*n* = 1223, 69%) prospectively recorded everything they had to eat and drink on four consecutive days into the web-based food record tool *Riksmaten2010* developed by the Swedish National Food Agency [31]. The food record tool aided estimation of portion sizes using photographs of foods of various portion sizes and was directly linked to the Swedish national food database. The intake of foods and nutrients was expressed as the average intake over the four recorded days. The intake of starch was calculated by subtracting the total sugar intake from the total carbohydrate intake, and it was investigated as the percentage of total nonalcoholic energy intake (E%) and divided into tertiles: < 24.9E%, 24.9–29.4E% and > 29.4E%. Fiber intake was examined as fiber density in g/1000 kcal. Participants also filled out a short food frequency questionnaire covering the preceding 6 months, from which the variable for bread intake (servings/week) was obtained (*n* = 1408, 80%).

#### Microbiome data

Participants were instructed on how to collect stool samples at home and store them in their home freezer until bringing them to their second visit at the research clinic where the samples were frozen at − 80 °C. Bacterial DNA was extracted with the QIAmp column stool kit, and sequencing of 16S rRNA (V1-V3 region) was performed with the HiSeq Illumina platform at GATC Biotech (Germany). QIIME was used to bin the sequences to operational taxonomic units that were matched to the Greengenes database (version 13.8). A total of 542 operational taxonomic units at the genus level were extracted from Greengenes. Operational taxonomic units that were identified in ≤ 2 individuals or with an abundance less than 0.01% were excluded, resulting in 64 included bacteria characterized at the genus level. Cumulative sum scaling in MetagenomicSeq in R was used to normalize the absolute abundances of the bacterial genera. The overall statistical analyses of gut microbiota were performed on 1412 (80%) participants who had data on both gut microbiota and *AMY1* CN, and stratified analyses were performed on 1038 (59%) participants who also had data on starch intake.

#### Statistical analysis

Statistical analyses were carried out using SPSS (Version 25; IBM Corporation, Armonk, NY, USA), except for microbiota analyses where Stata/SE was used (Version 15; StataCorp LLC, USA). Crude mean values and standard deviations (SD) are shown for baseline characteristics across groups of *AMY1* CN (categorized into four different groups: ≤ 4, 5–6, 7–9, ≥ 10) and across tertiles of starch intake. The differences between means for continuous baseline variables were analyzed across the *AMY1* CN groups and starch tertiles using one-way ANOVA. The differences between frequencies for categorical baseline variables were analyzed using the chi-squared test. A general linear model adjusted for age, sex and ethnicity (both/at least one/no parents born in Sweden or Denmark) was used to test the associations between *AMY1* CN (continuous variable) and fasting glucose and BMI. The analysis with fasting glucose was performed both with and without participants having a fasting plasma glucose > 6.1 mmol/L (prediabetes) (*n* = 156). To test the interaction effect between *AMY1* CN and starch intake on fasting glucose and BMI, an interaction term with *AMY1* × starch intake (both as continuous variables) was added to the model in addition to the main effects, adjusted according to two different models: age, sex, and ethnicity (basic model), and additionally including energy intake, smoking, and physical activity levels (fully adjusted model). The associations between *AMY1* CN (continuous) and outcomes were analyzed in strata of starch intake tertiles adjusted for age, sex and ethnicity, and the associations between starch intake (continuous) and outcomes were analyzed in strata of *AMY1* CN groups, adjusted for both the basic and fully adjusted model. A *P* value of < 0.05 was considered statistically significant.

Negative binomial regression adjusted for age, sex, and ethnicity was used to study the associations between *AMY1* CNV (continuous) and the abundance of 64 gut bacterial genera. To test the interaction effect between *AMY1* CN and starch intake on gut microbiota composition, an interaction term with *AMY1* × starch intake (both as continuous variables) was added to the regression models, first adjusting for age, sex, and ethnicity and additionally adjusting for energy intake, smoking, and physical activity levels. These analyses were also performed in strata of starch intake tertiles. This analysis was further explored with an additional adjustment for fiber intake. A false discovery rate (FDR) of 5% using the Benjamini-Hochberg method was used to correct for multiple testing [32].

### Study 2—Postprandial responses after two meals containing different starch doses

The study was approved by the regional ethics committee in Lund (Dnr 2018/968) and registered on ClinicalTrials.gov, identifier: NCT03974126. Prior to the study, all participants gave written informed consent.

#### Study sample

Using genotyped-based recall, participants in the MOS study who had been previously genotyped in 2016 for *AMY1* (*n* = 436) were recruited in spring 2019. Participants were selected from the extreme groups: those with ≤ 4 (*n* = 86) and ≥ 10 *AMY1* CN (*n* = 87). Using data from the MOS baseline, we excluded current smokers, individuals with diabetes, individuals with fasting blood glucose above 6.1 mmol/L, individuals with celiac disease, and individuals with missing contact information (*n* = 54 in total). Invitations to participate in the meal study were sent to 119 individuals. Before enrollment, it was ensured that participants were not current smokers, did not use medications known to influence glucose metabolism, appetite, or saliva flow, were not allergic to wheat, did not have celiac disease, were not on a low-carbohydrate diet, had not been diagnosed with diabetes since baseline examination in MOS, or were unable to consume up to 4 slices of white wheat bread in 15 min. A total of 23 individuals were enrolled in the meal study; however, 3 participants dropped out and 1 participant was excluded from analysis due to breastfeeding during the study. In total, 9 participants with low (≤ 4 copies) *AMY1* CN and 10 participants with high (≥ 10) *AMY1* CN completed the study.

#### Study design

The meal study had a crossover design with participants randomly allocated to a breakfast meal consisting of decorticated commercial white wheat bread (Jättefranska, Pågen AB, Malmö) providing 40 g or 80 g of starch and 250 ml of water on two separate test occasions, with a washout period of at least 7 days between visits. The starch content of the bread was determined using an enzymatic calorimetric method [33], from which we calculated the amount of bread to provide 40 g or 80 g of starch, resulting in 85 g and 170 g of bread, respectively. The meal tests were performed after a minimum of 11 h overnight fasting. Participants’ weight was recorded at fasting at the beginning of each visit. Participants were instructed to eat their breakfast meal at a steady pace over 15 min. Fingertip capillary blood samples were collected and measured at 0 (fasting), 7, 15, 30, 45, 60, 90, and 120 min after the start of the breakfast meal. Subjective appetite-related ratings covering satiety, hunger and desire to eat were determined at 0, 15, 30, 45, 60, 90, and 120 min with the use of a 100 mm visual analog scale (VAS). Participants were also asked to record how many times they chewed each of the first 5 standard bite-sized pieces of bread.

#### Analyses of biological samples

Capillary blood glucose was measured immediately using a HemoCue Glucose monitor (HemoCue AB, Ängelholm, Sweden). Blood for insulin analysis stood at room temperature for 30 min before being centrifuged for 10 min to separate the plasma portion to be stored at − 40 °C. Plasma insulin concentrations were determined using enzyme-linked immunosorbent assay (Mercodia Insulin ELISA, Mercodia, Uppsala, Sweden) in which samples were pipetted in duplicate and internal and external controls were used. Absorbance was read using a Tecan M200 Infinite Pro Reader (Tecan, Switzerland). To measure salivary α-amylase activity, fasting saliva samples were collected during the first visit with the use of a saliva collection tube (Salivette, Sarstedt AG & Co, Numbrecht, Germany), centrifuged and stored at − 20 °C. Activity was measured using an enzymatic assay (1-1902; Salimetrics, State College, PA). On the day of analysis, saliva samples were thawed, centrifuged, and diluted to a 1:200 dilution. Absorbance was measured at 405 nm at 1 and 3 min using a Tecan M200 Infinite Pro Reader (Tecan, Switzerland). Activity was calculated as the change in absorbance from 1 to 3 min. Absorbances were converted to units of activity/ml of salivary α-amylase as indicated by the kit producer.

#### Statistical analyses

Statistical analyses were carried out on GraphPad Prism (Version 8.2.0, GraphPad Software Inc. La Jolla, CA, USA). Due to the small sample size, differences between the groups in baseline characteristics and study results were analyzed nonparametrically using the Mann-Whitney *U* test. However, differences in incremental area under the curve (iAUC) between groups were analyzed both parametrically and nonparametrically using Student’s *t* test to facilitate comparison with previous studies. The iAUC was calculated for glucose and insulin as net change from baseline using the trapezoidal method. The total iAUCs are also displayed in boxplots for each *AMY1* CN group and starch dose. Each individual’s mean values (mm) over 120 min of VASs, which rated satiety, hunger, and desire to eat, were compared between the low and high *AMY1* CN groups for each starch dose. Likewise, the mean number of chews for the first 5 ingested pieces of bread was compared between groups. The incremental postprandial glucose and insulin values were further analyzed using three-way repeated measures ANOVA to determine the interactions between *AMY1* CN groups, starch dose and postprandial time, where time and starch dose were treated as matched measurements, as well as using two-way ANOVA separated by starch dose. A two-tailed *P* value of < 0.05 was considered statistically significant.

## Results

### Study 1—Cross-sectional cohort analysis of associations of *AMY1* CNV with fasting plasma glucose and BMI

Supplementary Figure 1 (Additional File 1) shows the participant flow throughout study 1 and study 2. In study 1, we included 1764 individuals (52% females) with a mean age of 39 years (range 18–71 years), BMI of 25.6 kg/m^2^, and *AMY1* CN of 6.7 (range 2–18). The mean (SD) reported starch intake was 27.3 (5.9) E%. No significant differences were observed in baseline characteristics across the *AMY1* CN groups (Table 1). There were fewer females in the higher starch intake groups, and individuals with high starch intake had a higher intake of fiber and bread (Table 2).

**Table 1:** Baseline characteristics of the study population across *AMY1* CN groups (*n* = 1764)

	*AMY1* CN groups				
	≤ 4 (*n* = 347)	5–6 (*n* = 676)	7–9 (*n* = 460)	≥ 10 (*n* = 281)	*P* value^1^
*AMY1* CN	3.65 (0.66)	5.71 (0.46)	7.92 (0.68)	11.01 (1.54)	
Females, *N*%	174 (50.1)	359 (53.1)	235 (51.1)	155 (55.2)	0.57
Age, years	38.5 (13.9)	39.5 (13.5)	38.7 (14.1)	37.4 (13.6)	0.17
BMI, kg/m^2^	25.5 (4.2)	25.4 (4.4)	25.9 (4.9)	25.3 (4.5)	0.25
Prediabetes, *N*%	25 (7.2)	55 (8.1)	49 (10.7)	27 (9.6)	0.30
Smokers, *N*%^3^	43 (14.3)	92 (15.7)	66 (16.5)	52 (21.1)	0.38
Parent born outside Swe/Den, *N*%	29 (8.4)	47 (7.0)	30 (6.5)	17 (6.0)	0.54
Physical activity level^2^	1.67 (0.13)	1.67 (0.14)	1.67 (0.14)	1.67 (0.13)	0.97
Total energy intake, kcal/day^2^	2015 (598)	2072 (919)	2044 (662)	1985 (686)	0.55
Starch intake, E%^2^	27.1 (5.2)	27.4 (6.0)	27.2 (6.3)	27.9 (5.5)	0.46
Fiber intake, g/1000 kcal^2^	9.50 (3.09)	9.94 (3.20)	9.61 (3.48)	9.57 (3.27)	0.26
Total bread intake, servings/week^4^	11.4 (7.8)	11.8 (8.2)	11.7 (8.8)	10.6 (7.2)	0.31

Values are unadjusted means (SD) unless stated otherwise

^1^*P* values were determined using one-way ANOVA for continuous variables and the chi-square test for categorical variables

^2^Data from 4-day food record, *n* = 1223

^3^Data available for *n* = 1531

^4^Data from FFQ, *n* = 1408

*AMY1*, salivary α-amylase gene; CN, copy number

**Table 2:** Baseline characteristics of the study population across tertiles of starch intake (*n* = 1223)

	Starch intake tertiles			
	Low < 24.9 E% (*n* = 407)	Medium 24.9–29.4 E% (*n* = 408)	High > 29.4 E% (*n* = 408)	*P* value^1^
Starch intake, E%	21.3 (3.7)	27.3 (1.3)	33.5 (3.7)	
*AMY1* CN	6.7 (2.5)	6.6 (2.6)	6.9 (2.5)	0.24
Females, *N*%	255 (62.7)	223 (54.7)	186 (45.6)	< 0.01
Age, years	39.5 (13.6)	39.6 (13.6)	39.5 (14.1)	1.00
BMI, kg/m^2^	25.5 (4.8)	25.4 (4.4)	25.3 (4.2)	0.86
Prediabetes, *N*%	40 (9.8)	31 (7.6)	36 (8.8)	0.53
Smokers, *N*%^2^	52 (13.8)	45 (11.9)	57 (15.2)	0.10
Physical activity level	1.67 (0.13)	1.67 (0.14)	1.66 (0.14)	0.49
Parent born outside Swe/Den, *N*%	22 (5.4)	23 (5.6)	31 (7.6)	0.65
Total energy intake, kcal/day	2076 (660)	2070 (669)	1971 (922)	0.09
Fiber intake, g/1000 kcal	8.6 (3.1)	9.5 (2.8)	11.0 (3.4)	< 0.01
Total bread intake, servings/week^3^	9.9 (7.1)	12.1 (8.2)	12.4 (8.8)	< 0.01

Values are unadjusted means (SD) unless stated otherwise

^1^*P* values were determined using one-way ANOVA for continuous variables and the chi-square test for categorical variables

^2^Data from *n* = 1130

^3^Data from FFQ, *n* = 1089

*AMY1*, salivary α-amylase gene; CN, copy number

No significant associations were observed between *AMY1* CNV and fasting plasma glucose or BMI (Table 3). However, a significant negative interaction was observed between *AMY1* CN and starch intake on fasting plasma glucose (*P* = 0.03) and BMI (*P* = 0.05) after full covariate adjustment. Since full adjustment limited the sample size to *n* = 1130, we also performed the interaction analyses with basic adjustment, and similar interaction effects were observed (glucose *P* = 0.02 and BMI *P* = 0.07). When stratifying into tertiles of starch intake, *AMY1* CN was negatively associated with fasting glucose in the high-starch group (*ß* = − 0.02 per CN, *P* = 0.06) and positively associated with fasting glucose in the low-starch group (*ß* = 0.02 per CN, *P* = 0.06). When stratifying into *AMY1* groups, a tendency for an inverse association between starch intake and BMI was observed in the highest *AMY1* CN groups (*ß* = − 0.09, *P* = 0.10) after full covariate adjustment. Overall, the highest fasting glucose concentration was observed in the group with ≥ 10 *AMY1* CN with the lowest starch intake*.* In addition, when individuals with fasting glucose concentrations above 6.1 mmol/L were included, the association between *AMY1* CNV and fasting plasma glucose was stronger (*ß* = 0.014, *P* = 0.04), but the interaction effect was similar (*P* = 0.02) (Supplementary Table 1, Additional File 1).

**Table 3:** Fasting plasma glucose and BMI across *AMY1* CN groups and in strata of starch intake tertiles

	*AMY1* CN groups				*Per AMY1* CN		Starch interaction			
	≤ 4	5–6	7–9	≥ 10	*ß*	*P*	*ß*^1^	*P*^1^	Adjusted *ß*^2^	Adjusted *P*^2^
**Fasting plasma glucose (excl individuals with prediabetes)**										
All (*n* = 1608)	5.19 (5.13–5.24)	5.20 (5.16–5.25)	5.21 (5.16–5.27)	5.25 (5.19–5.31)	0.004	0.40	− 0.002	0.02	− 0.002,	0.03
Low starch < 24.9E% (*n* = 367)	5.17 (5.05–5.29)	5.13 (5.03–5.22)	5.20 (5.09–5.32)	5.31 (5.17–5.45)	0.017	0.06				
Medium starch 24.9–29.4 E% (*n* = 377)	5.11 (4.98–5.23)	5.21 (5.10–5.32)	5.21 (5.09–5.34)	5.19 (5.05–5.34)	0.004	0.68				
High starch > 29.4E% (*n* = 372)	5.24 (5.11–5.37)	5.24 (5.14–5.34)	5.16 (5.05–5.27)	5.16 (5.03–5.29)	− 0.017	0.06				
*ß* per E% of starch^3^	0.005	0.006	− 0.009	− 0.009						
*P* value^2^	0.43	0.08	0.09	0.14						
Adjusted *ß* per E% of starch^4^	0.005	0.007	− 0.011	− 0.004						
Adjusted *P* value^4^	0.42	0.07	0.05	0.47						
**BMI**										
All (*n* = 1764)	25.3 (24.8–25.8)	25.2 (24.8–25.6)	25.7 (25.3–26.2)	25.3 (24.7–25.9)	0.05	0.26	− 0.017	0.07	− 0.018	0.05
Low starch < 24.9E% (*n* = 407)	25.4 (24.2–26.6)	24.6 (23.7–25.6)	25.6 (24.5–26.8)	25.5 (24.1–26.8)	0.08	0.39				
Medium starch 24.9–29.4 E% (*n* = 408)	24.7 (23.7–25.8)	25.1 (24.2–26.0)	26.0 (24.6–27.0)	25.8 (24.6–27.0)	0.18	0.03				
High starch > 29.4E% (*n* = 408)	25.5 (24.5–26.6)	25.1 (24.3–25.9)	25.2 (24.3–26.1)	24.3 (23.2–25.4)	− 0.14	0.09				
*ß* per E% of starch^3^	− 0.03	− 0.02	− 0.07	− 0.11						
*P* value^2^	0.57	0.57	0.12	0.04						
Adjusted *ß* per E% of starch^4^	− 0.03	0.002	− 0.06	− 0.09						
Adjusted *P* value^4^	0.53	0.96	0.19	0.10						

Values are presented as the means (95% CI) adjusted for age, sex, and ethnicity unless stated otherwise

^1^Interaction *AMY1* CN × starch intake, both as continuous variables, adjusted for age, sex and ethnicity (*n* = 1223, excluding individuals with prediabetes *n* = 1116)

^2^Interaction *AMY1* CN × starch intake, both as continuous variables, adjusted for age, sex, and ethnicity, energy intake, smoking habits, and physical activity level (*n* = 1130, excluding individuals with prediabetes *n* = 1026)

^3^Association between starch intake (continuous) and fasting glucose or BMI in strata of *AMY1* CN adjusted for age, sex, and ethnicity.

^4^Association between starch intake (continuous) and fasting glucose or BMI in strata of *AMY1* CN adjusted for age, sex, ethnicity, energy intake, smoking habits, and physical activity level

*AMY1*, salivary α-amylase gene; CN, copy number

No significant association was seen between *AMY1* CN (as a continuous variable) and any of the bacterial genera after adjusting for multiple testing using FDR (Supplementary Table 2, Additional File 1). We then tested for interaction with habitual starch intake and observed a nominally significant effect of interaction between *AMY1* CN and starch intake on two out of 64 genera, *Megasphaera* (*P* = 0.01) and *Acidaminococcus* (*P* = 0.05), and found similar results when this was analyzed with further adjustments for energy intake, smoking habits, and physical activity level. When stratifying into tertiles of starch intake, no significant associations were seen for *Megasphaera* and *Acidaminococcus* in any starch group, but nominal associations were seen among those with high starch intake with *Paraprevotella* (*β* = 0.06, *P* = 0.04). However, no associations remained significant after adjusting for multiple testing using an FDR of 0.05. In an additional analysis, adding fiber intake as a covariate to the model did not markedly affect the results.

### Study 2—Postprandial responses after two meals containing different starch doses

Median fasting salivary α-amylase activity was more than four times higher in those with high (≥ 10 copies) *AMY1* CN (range 70–156 U/ml) than in those with low (≤ 4 copies) *AMY1* CN (range 8–53 U/ml). Otherwise, participants did not differ in baseline characteristics (Table 4).

**Table 4:** Participant characteristics in the meal study (*n* = 19)

	Low *AMY1* CN (*n* = 9)	High *AMY1* CN (*n* = 10)	*P* value^2^
*AMY1* CN^1^	4 (2–4)	11 (10–16)	
Salivary α-amylase activity, U/mL	23.0 (7.7–52.7)	107.6 (69.6–155.9)	< 0.01
Females, *n* (%)	5 (56%)	4 (40%)	0.65^3^
Parent born outside Swe/Den, *n* (%)	1 (11%)	1 (10%)	1.00^3^
Age, years	48.0 (28.6–57.6)	58.4 (37.8–66.7)	0.07
BMI¸ kg/m^2^	28.0 (21.9–29.9)	26.9 (23.4–28.7)	0.74
Fasting capillary blood glucose, mmol/L	5.35 (5.20–5.55)	5.08 (4.83–5.55)	0.17
Fasting plasma insulin, pmol/L	49.1 (37.7–63.0)	48.3 (37.3–69.5)	0.97
Habitual total bread intake, servings/week^4^	9.57 (6.45–10.47)	7.45 (5.64–11.04)	0.65
Habitual white bread intake, servings/week^4^	1.07 (0.63–1.5)	1.07 (0.35–5.5)	0.79

Values are presented as medians (IQRs) unless stated otherwise

^1^Values are presented as median (range)

^2^*P* values were determined with the Mann-Whitney *U* test unless stated otherwise

^3^*P* value is determined with Fisher’s exact test

^4^From MOS baseline FFQ, *n* = 8 for both groups

*AMY1*, salivary α-amylase gene; CN, copy number; FFQ, food frequency questionnaire; MOS, Malmö Offspring Study

**Fig. 1:**
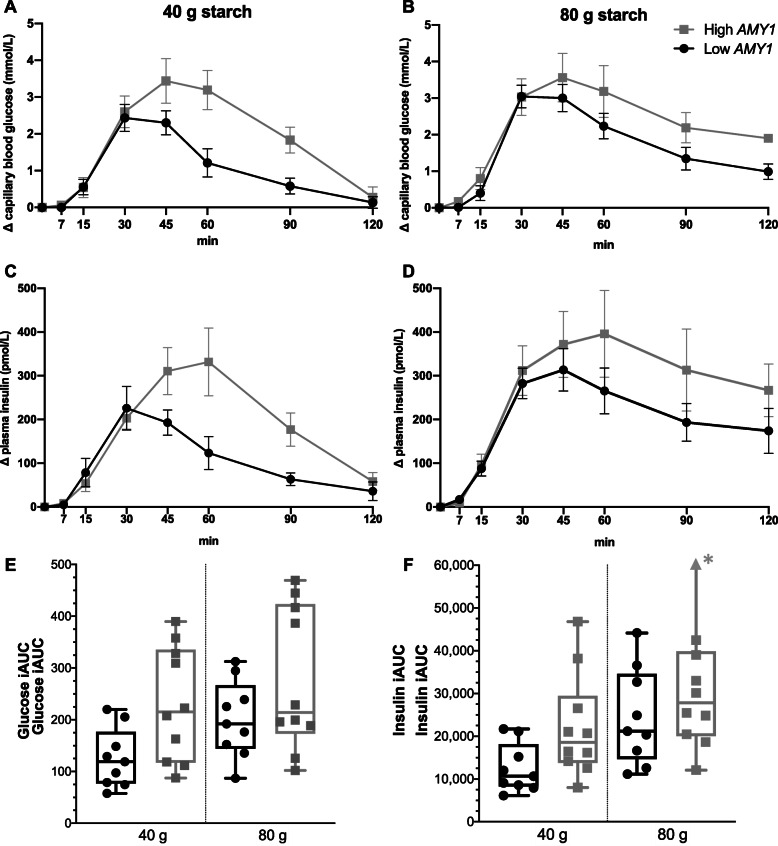
Change in postprandial response (mean ± SE) among the low *AMY1* CN group (*n* = 9, black) and high *AMY1* CN group (*n* = 10, gray) over time (min) after a starch meal. **A** Capillary blood glucose response to 40 g starch, **B** capillary blood glucose response to 80 g starch, **C** plasma insulin response to 40 g starch, and **D** plasma insulin response to 80 g starch. Three-way ANOVA demonstrated a significant *AMY1* CN group × time interaction (*P* = 0.04) and *AMY1* CN group × starch dose × time interaction (*P* = 0.02) for glucose. For insulin, only the *AMY1* CN group × time interaction was borderline significant (*P* = 0.06). Two-way ANOVA separated by starch dose demonstrated significant *AMY1* CN group × time interactions for both glucose and insulin at 40 g (*P* = 0.0011 and *P* = 0.0007) but not at 80 g (*P* = 0.49 and *P* = 0.59), respectively. Boxplots of the total iAUC for each group and starch dose are displayed in **E** for glucose and **F** for insulin. *Whisker reaches iAUC 91,211. *AMY1*, salivary α-amylase gene; CN, copy number; iAUC incremental area under the curve

Following ingestion of 40 g starch, the high *AMY1* CN group had an 83% higher iAUC for postprandial glucose (*P* = 0.02) (Fig. 1A) and a 73% higher iAUC for insulin (*P* = 0.05) (Fig. 1C) compared to the low *AMY1* CN group (Table 5). Following the ingestion of 80 g starch, the differences between the *AMY1* CN groups were smaller and nonsignificant; the high *AMY1* CN group had a 38% higher iAUC for postprandial glucose (*P* = 0.17) (Fig. 1B) and a 37% higher iAUC for insulin (*P* = 0.07) (Fig. 1D). Within both the low and high *AMY1* groups, the iAUCs for both glucose and insulin were significantly higher after 80 g starch than after 40 g starch. However, mean individual differences in glucose (*P* = 0.33) and insulin (*P* = 0.28) responses after 40 and 80 g starch did not differ between the low and high *AMY1* groups (Table 5). These results were similar when analyzed using the Mann-Whitney *U* test instead of Student’s *t* test (Supplementary Table 3, Additional File 1). The variations in glucose and insulin responses were consistently larger in the high *AMY1* CN than in the low *AMY1* CN group, as displayed in the boxplots in Fig. 1E, F. Furthermore, for glucose, there appeared to be a separation, where four individuals in the high *AMY1* CN group had a larger response following both 40 g and 80 g of starch. However, we could not identify any clear variation in either *AMY1* CN, amylase activity, age, sex, or BMI to explain this separation.

**Table 5:** Postprandial responses for capillary blood glucose and plasma insulin following consumption of 40 g and 80 g starch (*n* = 19)

	Low *AMY1* CN (*n* = 9)	High *AMY1* CN (*n* = 10)	*P* value^1^
**Blood glucose, mmol/L**			
iAUC 40 g	125.6 (57.1)	229.7 (110.4)	0.02
iAUC 80 g	201.7 (73.8)	275.8 (138.6)	0.17
*P* value^2^	0.02	0.02	
Individual differences in iAUC between 40 g and 80 g	76.0 (75.7)	46.1 (54.1)	0.33
**Plasma insulin, pmol/L**			
iAUC 40 g	12,486 (5685)	22,056 (12,076)	0.05
iAUC 80 g	24,465 (11,235)	33,729 (22,216)	0.07
*P* value^2^	0.005	0.02	
Individual differences in iAUC between 40 g and 80 g	11,979 (9246)	11,673 (13,706)	0.28

Values are presented as the mean (SD)

^1^*P* value testing differences between *AMY1* groups are determined with unpaired *t* tests

^2^*P* value testing differences between doses within *AMY1* groups are determined with paired *t* tests

*AMY1*, salivary α-amylase gene; CN, copy number, iAUC, incremental area under the curve

From three-way ANOVA analyses, the main effect of the *AMY1* CN groups on postprandial glucose was not significant (*P* = 0.09), but we identified a significant *AMY1* CN group × time interaction (*P* = 0.04) and *AMY1* CN group × starch dose × time interaction (*P* = 0.02). For insulin, only the *AMY1* CN group × time interaction was borderline significant (*P* = 0.06). Two-way ANOVA separated by starch dose showed that the *AMY1* CN group × time interactions were significant for both glucose and insulin at 40 g (*P* = 0.0011 and *P* = 0.0007) but not at 80 g (*P* = 0.49 and *P* = 0.59, respectively). There were significant differences at specific time points between the *AMY1* groups for postprandial glucose at 60 and 90 min for 40 g starch and 120 min for 80 g starch (Supplementary Table 4, Additional File 1). Significant differences were also seen between the groups for insulin at 45, 60, and 90 min for 40 g starch. The average number of chews and VAS ratings for hunger, satiety, and desire to eat did not differ between the *AMY1* groups for either 40 g or 80 g starch intake (Supplementary Table 5, Additional File 1).

## Discussion

In a cohort of 1223 individuals of largely homogenous Swedish descent, an interaction effect between *AMY1* CN and habitual starch intake on fasting glucose and BMI was observed. *AMY1* CN was inversely associated with fasting glucose and BMI at a high starch intake level but positively associated at low starch intake level. In our meal study, we also observed significantly higher postprandial glucose and insulin levels in the group with high *AMY1* CN than in that with low CN when consuming 40 g starch but not 80 g starch.

No overall association between *AMY1* CN and fasting plasma glucose or BMI was found in our cross-sectional cohort analysis. The lack of an association with BMI in MOS resembles results from other cohorts [15–22], including the study with the parents and grandparents of MOS participants (the MDC-CC) [25]. However, other studies have shown negative associations between *AMY1* CNV and BMI [5–14]. These discrepancies may be due to methodological differences, specifically genotyping methods [15], or to different dietary habits in the different populations. In both the MOS and MDC-CC cohorts, starch intake seemed to modify the association between *AMY1* CN and BMI [25], although in opposite directions. Two additional studies have identified an interaction with starch intake. Vazquez-Moreno et al. demonstrated an interaction with the odds of childhood obesity in the same direction as that in the present study, but only when studying serum amylase activity and not *AMY1* CN [20]. Heianza et al. [34] found an interaction between an *AMY1* genetic risk score and carbohydrate food intake on BMI changes in the same direction as that in MDC-CC [25]. This discrepancy in the direction of the interactions indicates the need for replication in other cohorts.

Few studies have investigated the association between *AMY1* CNV and measures of glucose homeostasis [9, 11, 12, 16, 18, 21]. Similar to that on BMI, we observed an interaction effect between starch intake and *AMY1* CN on fasting plasma glucose levels. Only at a high starch intake level was *AMY1* CN inversely associated with BMI and fasting glucose. Actually, the highest fasting glucose level was seen among those with the highest *AMY1* CN and lowest starch intake in this study. This interaction resembles the results seen in MDC-CC for fasting insulin levels and HOMA-IR, where the highest levels were also seen with the highest *AMY1* CN and lowest starch intake and a positive association with *AMY1* CN was limited to low starch consumers. However, no interaction with fasting glucose was observed in MDC-CC [23]. It may be suggested that individuals with high *AMY1* CN have adapted to digest and absorb starch more efficiently. In addition, those with high starch intake had high fiber intake, indicating a healthier overall dietary pattern. Notably, this population on average did not have a very high starch intake (27.3E%). Nevertheless, although *AMY1* CN is associated with postprandial glucose, the same should not be anticipated for fasting glucose, as long-term glucose homeostasis is tightly regulated in healthy individuals [35, 36].

It has been suggested that *AMY1* CN may influence the gut microbiota composition. Those with low *AMY1* CN may be unable to fully digest starch; therefore, a fraction of the consumed starch may reach the colon and be fermented by gut bacteria. Three other studies have investigated the relationship between *AMY1* CN and the gut microbiota [11, 21, 24]. Barber et al. found no association between *AMY1* CN and the gut microbiota [21]. Leon-Mimila et al. found a positive correlation between *AMY1* CN and *Prevotella* abundance at the genus level in a Mexican population of 45 adults [11]. Poole et al., with a population of approximately 1000 participants, found that members of the *Ruminococcaceae* family were enriched in high *AMY1* CN individuals [24]. Both *Ruminococcaceae* and *Prevotella* have been linked to the gut fermentation of resistant starch [37–39]. These positive associations contradict the hypothesis of more undigested starch reaching the colon in low *AMY1* CN individuals. On the other hand, sixfold higher breath methane levels following starch ingestion in those with low *AMY1* CN have also been reported [18]. We did not find any association between *AMY1* CNV and gut microbiota in our study, despite a larger sample size than those of the previous studies. However, our gut microbiota analysis was limited to genomic sequencing of the 16S rRNA gene. Whole metagenomic analysis may help clarify the relationship between *AMY1* CN and the gut microbiota.

We found 83% higher postprandial glycemia in those with ≥ 10 copies of *AMY1* than in those with ≤ 4 copies after ingestion of 40 g starch from white bread, suggesting an increased ability to absorb glucose from starch. This agrees partly with results by Atkinson et al., who found 16% higher glycemia after 50 g carbohydrate from white bread in 20 participants with ≥ 10 copies than in 20 participants with ≤ 4 copies [18]. However, Higuchi et al. found no difference in postprandial glycemia after 130 g rice (ca 35 g starch) when comparing *AMY1* CN 4-7 with *AMY1* CN 8-14 (*n* = 60) [26]. Furthermore, our results conflict with those found by Mandel and Breslin using a 50 g starch solution in a study of 10 volunteers [27] and Barling et al. using 101.8 g white bread (ca 44 g starch) in a study of 14 volunteers [28], both showing a lower glucose response among those with high salivary α-amylase activity. However, these studies did not investigate *AMY1* CN. The advantage of using *AMY1* CN is that it remains stable throughout life, whereas salivary α-amylase activity can vary greatly among individuals on a day-to-day basis [40].

A 77% higher insulin response was recorded in those with high *AMY1* CN than in those with low *AMY1* CN after ingestion of 40 g starch, a difference that was not observed in the study by Atkinson et al. [18]. However, Mandel and Breslin found that the cephalic phase of insulin secretion was increased in those with high salivary amylase activity [27]. We failed to find differences during the cephalic phase (7 min measurement), perhaps because participants had consumed only half of the starch dose by then, and bread requires longer digestion than liquid starch solutions.

To our knowledge, 80 g is the largest dose of starch examined so far in comparing the postprandial response according to *AMY1* CNV. It corresponds to approximately 5 slices of white bread or 300 g cooked pasta, which is a large but still realistic starch dose for one meal. Contrary to our expectations, the difference was smaller and nonsignificant between the two groups’ postprandial responses when consuming 80 g starch. Insufficient statistical power due to the small sample size could have contributed to this finding, as Fig. 1 visually suggests differences between *AMY1* CN groups also at 80 g. A saturation effect could also be suggested, i.e., after a certain level of starch intake, both groups react similarly in terms of glucose and insulin responses, regardless of the *AMY1* CN. This interpretation should be tested with differing doses of starch, preferably with an intermediate dose of 60 g and a larger sample size, to clarify any saturation hypothesis. Furthermore, it cannot be ruled out that differences other than in *AMY1* CN may be present between the study groups as group allocation was based on genetics instead of randomization. For example, there appears to be an unfortunate age difference between the groups, which could have contributed to the higher postprandial glucose responses in the high *AMY1* CN group, perhaps contributing to the larger observed differences in postprandial glucose between the high and low *AMY1* CN groups in this study (83%) than in a previous study (16%) [18]. However, there were no differences in fasting plasma glucose and insulin between the groups, indicating that the general metabolic health did not differ between groups despite a slight age difference. An additional postprandial test following a glucose load could be useful in future studies to clarify to what extent the differences between groups following starch intake actually depend on their ability to digest starch. Nevertheless, these findings agree with the interaction between *AMY1* CN and starch intake identified in our cross-sectional observational study; at a high starch intake level, high *AMY1* CN is not associated with increased fasting glucose.

The major limitation of the observational study is that it is limited to cross-sectional analyses of associations between *AMY1* CN and starch intake with BMI and fasting glucose. The strengths of our meal study include using genotype-based recall to recruit participants, having access to comprehensive data on participants from the MOS cohort and *AMY1* CN genotyped by ddPCR, referred to as the “gold standard” for determining *AMY1* CN [41]. Future studies should investigate the postprandial response to starchy foods for more than 120 min, since glucose and insulin did not return to baseline levels after consumption of 80 g of starch, as well as the long-term effect after several days or weeks. Furthermore, initiatives to identify traits that explain the large variation in response that was observed are warranted. If this can be elucidated, these findings could allow for personalized nutrition strategies to reduce obesity and type 2 diabetes by tailoring starch intake recommendations depending on patients’ *AMY1* CN.

## Conclusions

In conclusion, a diet-gene interaction between *AMY1* CN and starch intake was observed on fasting plasma glucose and BMI, suggesting that at high starch intake levels, *AMY1* CN associates inversely with fasting glucose and BMI, while the opposite was observed at low starch intake levels. Postprandial glucose and insulin responses also differed according to *AMY1* CN and starch dose. After ingestion of 40 g starch, glucose and insulin responses were reduced in low *AMY1* CN individuals. However, the differences were smaller and nonsignificant after the starch dose was doubled.

## Supplementary Information

**Additional file 1:.** Figure S1 and Tables S1-S5

## Data Availability

The datasets generated and/or analyzed during the current study are not publicly available due to ethical and legal restrictions but are available from the corresponding author on reasonable request (meal study data) or pending request and approval by the steering committee for the Malmö cohorts; see instructions at https://www.malmo-kohorter.lu.se/english (MOS cohort data).

## Abbreviations

*AMY1*: Salivary α-amylase gene
*AMY2*: Pancreatic amylase gene
BMI: Body mass index
CI: Confidence interval
CN: Copy number
CNV: Copy number variation
ddPCR: Droplet digital polymerase chain reaction
FDR: False discovery rate
iAUC: Incremental area under the curve
IQR: Interquartile range
MDC-CC: Malmö Diet and Cancer-Cardiovascular Cohort
MOS: Malmö Offspring Study
SD: Standard deviation
VAS: Visual analog scale
