# Supplementary material for: Effect of AMY1 copy number variation and various doses of starch intake on glucose homeostasis: data from a cross-sectional observational study and a crossover meal study
Source: Genes Nutr. 2021 Nov 17;16:21. doi: 10.1186/s12263-021-00701-8 (PMC8596830; doi:10.1186/s12263-021-00701-8)
Supplement: Supplementary file 1 — Additional file 1:. Figure S1 and Tables S1-S5 [file 12263_2021_701_MOESM1_ESM.pdf]

Additional File 1

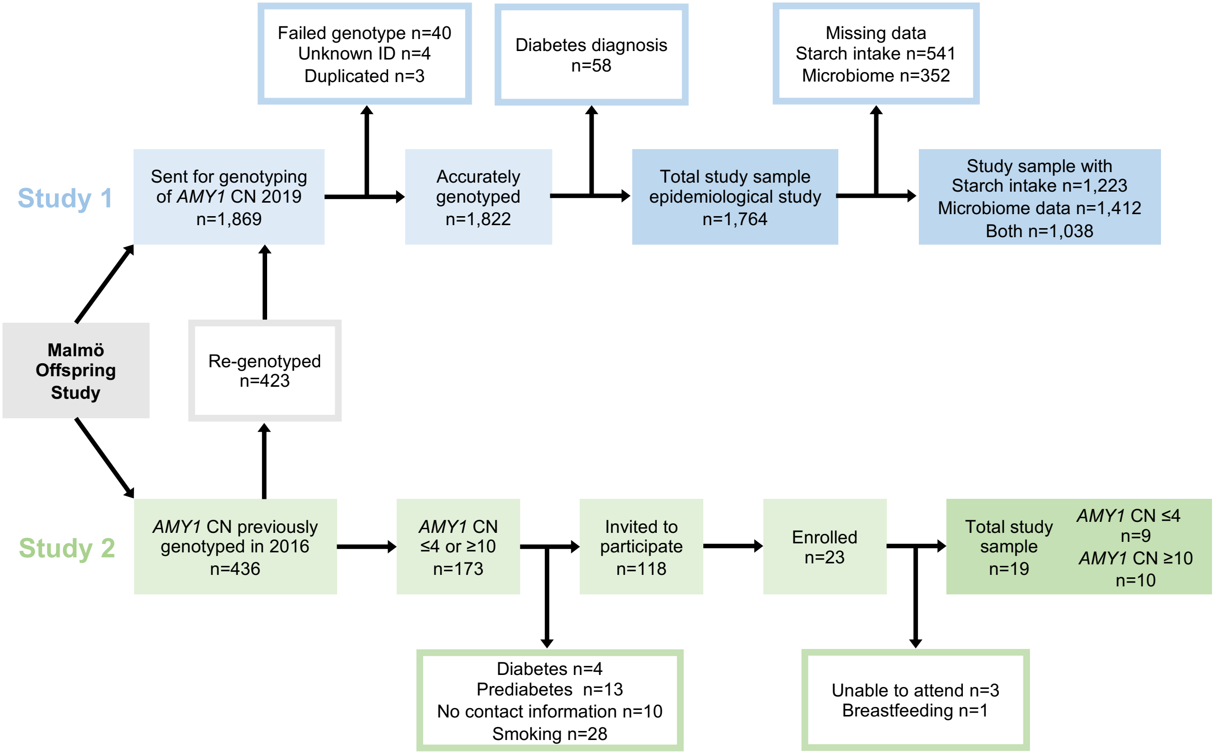

Supplementary Figure 1. Flowchart of participants for Study 1 and Study 2

Supplementary Table 1. Fasting plasma glucose across *AMY1* CN groups and in strata of starch intake tertiles in full study sample (n=1,764)[1]

	AMY1 CN groups				*Per AMY1* CN		Interaction	
	≤4	5-6	7-9	≥10	ß	*P*	ß	P^2
**Fasting plasma glucose (all)**								
All (n=1,764)	5.29 (5.22-5.37)	5.33 (5.26-5.39)	5.39 (5.32-5.46)	5.41 (5.32-5.49)	0.014	0.04	-0.003	0.02
Low starch <24.9E% (n=407)	5.26 (5.12-5.41)	5.18 (5.06-5.29)	5.33 (5.20-5.47)	5.41 5.25-5.58)	0.024	0.03		
Medium starch 24.9-29.4 E% (n=408)	5.22 (5.01-5.44)	5.33 (5.14-5.51)	5.54 (5.34-5.75)	5.46 (5.22-5.71)	0.036	0.03		
High starch >29.4E% (n=408)	5.33 (5.18-5.48)	5.35 (5.23-5.46)	5.26 (5.14-5.39)	5.25 (5.09-5.40)	-0.019	0.08		
ß per E% of starch[3]	0.005	0.009	-0.013	-0.006				
P-value[3]	0.51	0.02	0.05	0.33				
Adjusted ß per E% of starch[4]	0.004	0.010	-0.015	-0.001				
Adjusted *P*-value[4]	0.53	0.02	0.04	0.88				

[1] Values are presented as means (95% CI) adjusted for age, sex and ethnicity unless stated otherwise.
[2] Interaction *AMY1* CN×starch intake, both as continuous variables, adjusted for age, sex and ethnicity.
[3] Association between starch intake (continuous) and fasting glucose or BMI in strata of *AMY1* CN adjusted for age, sex and ethnicity.
[4] Association between starch intake (continuous) and fasting glucose or BMI in strata of *AMY1* CN adjusted for age, sex, ethnicity, energy intake, smoking habits and physical activity level.
AMY1, salivary α-amylase gene; CN, copy number

Supplementary Table 2. Association between *AMY1* CNV and 64 bacterial genera examined with negative binomial regressions[1].

	Continuous analysis			Stratified analysis					
	Overall association (n=1,412)		Starch interaction[2] (n=1038)	Low starch (n=358)		Medium starch (n=339)		High starch (n=341)	
	β	*P*	*P*	β	*P*	β	*P*	β	*P*
Megasphaera	0.039	0.096	**0.010**	-0.031	0.546	0.019	0.657	0.088	0.086
Parabacteroides	-0.005	0.127	0.572	-0.004	0.543	-0.004	0.506	0.002	0.793
Prevotella	0.011	0.149	0.724	0.010	0.490	0.023	0.132	-0.007	0.673
Paraprevotella	0.022	0.150	0.959	0.051	0.110	-0.004	0.907	0.061	**0.042**
Unknown genus in order of *Bacteroidales*	0.024	0.183	0.358	0.031	0.431	0.039	0.267	-0.014	0.722
Peptococcus	-0.059	0.205	0.844	-0.149	0.159	-0.131	0.138	-0.030	0.809
Dialister	0.008	0.213	0.173	0.016	0.206	-0.010	0.391	-0.010	0.476
Akkermansia	-0.009	0.243	0.201	0.012	0.480	-0.019	0.213	-0.025	0.131
Oxalobacter	0.017	0.253	0.677	0.041	0.226	0.011	0.705	0.023	0.444
Odoribacter	0.019	0.257	0.878	0.036	0.299	-0.007	0.837	0.030	0.402
Phascolarctobacterium	-0.010	0.282	0.542	-0.004	0.833	-0.013	0.452	0.003	0.873
Unknown genus in family of *RF16*	0.019	0.303	0.581	0.043	0.245	0.020	0.630	0.010	0.767
Unknown genus in family of *Coriobacteriaceae*	-0.004	0.311	0.898	-0.005	0.479	0.006	0.439	-0.004	0.611
[*Prevotella*]	-0.022	0.334	0.685	-0.051	0.320	-0.077	0.132	-0.062	0.165
Dorea	-0.003	0.335	0.859	-0.006	0.430	-0.002	0.803	-0.006	0.435
Unknown genus in order of *SHA98*	0.015	0.354	0.875	0.019	0.561	0.004	0.893	0.035	0.271
[*Ruminococcus*]	-0.003	0.360	0.931	-0.006	0.394	0.003	0.669	-0.008	0.283
Unknown genus in family of [*Mogibacteriaceae*]	0.004	0.360	0.564	-0.005	0.566	0.007	0.407	0.004	0.658
Veillonella	0.005	0.367	0.863	0.004	0.770	-0.002	0.880	-0.004	0.708
Lactococcus	0.007	0.390	0.760	-0.003	0.848	0.019	0.263	-0.001	0.948
Sutterella	-0.010	0.395	0.956	0.008	0.765	-0.016	0.505	-0.006	0.779
Unknown genus in family of *Enterobacteriaceae*	0.009	0.403	0.709	0.014	0.510	-0.018	0.379	0.023	0.248
Collinsella	-0.003	0.410	0.150	-0.009	0.285	-0.007	0.412	0.007	0.393
Unknown genus in order of *RF32*	-0.013	0.422	0.375	-0.007	0.829	-0.044	0.189	-0.017	0.592
[*Eubacterium*]	-0.005	0.443	0.525	-0.006	0.681	-0.024	0.108	0.009	0.538
Unknown genus in family of *Rikenellaceae*	-0.002	0.457	0.523	-0.005	0.431	-0.003	0.575	0.002	0.803
Unknown genus in family of *Clostridiaceae*	0.003	0.464	0.643	0.003	0.736	0.006	0.465	-0.005	0.568
Turicibacter	-0.005	0.467	0.206	0.002	0.901	-0.002	0.900	-0.014	0.375
Faecalibacterium	-0.002	0.506	0.892	-0.007	0.260	0.002	0.717	-0.007	0.243
SMB53f	0.004	0.513	0.148	0.016	0.163	-0.008	0.458	0.002	0.855
Catenibacterium	-0.018	0.529	0.524	-0.008	0.913	-0.071	0.248	-0.002	0.973
Unknown genus in family of *Christensenellaceae*	0.004	0.535	0.082	-0.014	0.303	0.015	0.206	0.020	0.133
Ruminococcus	-0.002	0.538	0.965	-0.007	0.316	0.002	0.727	-0.004	0.506
Fusobacterium	0.011	0.540	0.480	-0.001	0.980	-0.011	0.760	0.006	0.874
Lachnobacterium	-0.003	0.578	0.346	-0.021	0.057	0.021	**0.049**	-0.012	0.294
Alistipes	0.005	0.602	0.278	-0.006	0.743	-0.003	0.859	0.022	0.248
Lactobacillus	0.006	0.604	0.329	0.006	0.795	0.026	0.218	-0.001	0.954
Unknown genus in family of *Halanaerobiaceae*	-0.002	0.641	0.363	0.004	0.568	-0.005	0.493	-0.004	0.639
Bacteroides	-0.001	0.654	0.622	0.002	0.779	-0.001	0.806	-0.002	0.701
Coprococcus	-0.001	0.663	0.720	-0.005	0.421	0.004	0.530	-0.006	0.399
Anaerotruncus	-0.005	0.670	0.788	-0.004	0.863	-0.018	0.375	0.000	0.996
Succiniclasticum	0.037	0.684	0.796	0.113	0.530	0.005	0.980	-0.100	0.709
Streptococcus	-0.002	0.689	0.745	-0.004	0.562	0.005	0.476	-0.002	0.822

Unknown genus in order of *Bacteroidales*	-0.004	0.702	0.358	-0.013	0.499	-0.004	0.846	0.018	0.383
Desulfovibrio	0.007	0.729	0.080	-0.059	0.186	0.040	0.313	0.063	0.126
Clostridium	-0.001	0.734	0.833	-0.003	0.671	-0.001	0.927	-0.005	0.592
Adlercreutzia	0.001	0.754	0.395	-0.002	0.817	0.005	0.546	-0.002	0.818
Unknown genus in family of [*Barnesiellaceae*]	-0.001	0.757	0.375	0.000	0.947	-0.001	0.886	0.003	0.666
Acidaminococcus	0.008	0.763	**0.048**	-0.067	0.218	0.018	0.710	0.071	0.244
Butyricimonas	-0.002	0.770	0.505	0.002	0.854	-0.011	0.295	0.012	0.293
Cetobacterium	-0.078	0.804	0.619	-0.063	0.905	-0.203	0.587	-0.727	0.509
Unknown genus in family of [*Paraprevotellaceae*]	0.009	0.812	0.614	-0.107	0.163	0.053	0.469	-0.043	0.575
Unknown genus in family of *Lachnospiraceae*	-0.001	0.830	0.905	-0.004	0.495	0.003	0.627	-0.002	0.693
Unknown genus in order of *Clostridiales*	-0.001	0.833	0.614	-0.005	0.431	0.003	0.563	-0.001	0.853
Unknown genus in family of *Peptostreptococcaceae*	-0.001	0.834	0.141	0.002	0.835	-0.001	0.902	-0.001	0.876
Anaerostipes	-0.001	0.868	0.835	-0.003	0.800	-0.007	0.416	0.010	0.283
Unknown genus in family of *Erysipelotrichaceae*	0.001	0.871	0.776	-0.007	0.320	0.013	0.067	-0.001	0.888
Blautia	0.000	0.878	0.797	-0.005	0.417	0.002	0.753	0.001	0.939
Unknown genus in family of *Ruminococcaceae*	0.000	0.879	0.813	-0.003	0.569	0.002	0.652	-0.001	0.824
Lachnospira	0.001	0.888	0.844	-0.006	0.473	0.005	0.596	-0.004	0.651
Unknown genus in order of *RF39*	-0.002	0.906	0.973	-0.032	0.273	0.025	0.313	-0.002	0.945
Unknown genus in order of *YS2*	0.002	0.920	0.537	0.024	0.513	0.004	0.913	-0.002	0.967
Oscillospira	0.000	0.949	0.541	-0.004	0.549	0.003	0.604	0.001	0.863
Roseburia	0.000	0.971	0.639	-0.012	0.092	0.008	0.238	-0.010	0.191

[1] Negative binomial regressions are adjusted for age, sex and ethnicity

[2] *P*-interaction of *AMY1* CN×starch intake (both as continuous variables) determined with adjustment for age, sex, ethnicity, energy intake.

No *P*-value remained significant after adjusting for FDR of 0.05.

Supplementary Table 3. Postprandial responses for capillary blood glucose and plasma insulin following consumption of 40 g and 80 g starch (n=19)[1]

	Low *AMY1* CN (n=9)	High *AMY1* CN (n=10)	*P*-value[2]
**Blood glucose, mmol/L**			
iAUC 40 g	119.0 (76.8-177.0)	215.3 (116.8-335.6)	0.04
iAUC 80 g	192.0 (143.9-266.9)	214.0 (173.0-423.7)	0.32
Individual differences in iAUC between 40 g and 80 g	89.35 (28.6-128.3)	49.40 (4.2-92.6)	0.28
**Plasma insulin, pmol/L**			
iAUC 40 g	10,650 (8,247-18,167)	18,571 (13,752-29,442)	0.05
iAUC 80 g	21,186 (14,609-34,627)	27,797 (20,018-39,891)	0.36
Individual differences in iAUC between 40 g and 80 g	12,099 (4,826-18,455)	7,515 (2,921-17,831)	0.66

[1] Values are presented as median (IQR).

[2] *P*-values are determined with a Mann-Whitney U test

AMY1, salivary α-amylase gene; CN, copy number, iAUC, incremental area under the curve

Supplementary Table 4. Differences in delta postprandial glucose and insulin response for each time ponit between groups for 40 g starch and 80 g starch[1]

	40 g starch			80 g starch		
Time point (min)	Low *AMY1* CN (n=9)	High *AMY1* CN (n=10)	*P*-value[2]	Low *AMY1* CN (n=9)	High *AMY1* CN (n=10)	*P*-value[2]
**Glucose**						
7	0.0 (-0.25-0.3)	0.10 (-0.23-0.3)	0.73	0.10 (-0.20-0.15)	0.20 (0.05-0.33)	0.09
15	0.30 (0.20-1.15)	0.15 (0.10-0.83)	0.39	0.50 (-0.10-0.65)	0.60 (0.15-1.43)	0.41
30	2.10 (1.50-3.40)	2.40 (2.05-3.28)	0.65	3.00 (2.45-3.70)	2.90 (1.33-4.55)	0.92
45	2.00 (1.75-2.75)	3.40 (2.03-4.95)	0.12	2.80 (2.10-3.85)	2.65 (1.98-5.70)	0.86
60	1.4 (0.05-2.35)	3.40 (1.73-4.45)	0.02	2.00 (1.45-3.10)	2.85 (1.25-5.18)	0.43
90	0.50 (0.25-0.65)	1.90 (0.93-2.45)	<0.01	1.20 (0.60-2.20)	2.20 (1.05-3.23)	0.16
120	0.00 (-0.25- 0.40)	0.50 (-0.13-0.73)	0.29	1.20 (0.45-1.50)	1.85 (1.68-2.10)	<0.01
**Insulin**						
7	5.15 (-7.53-15.57)	7.76 (-8.78-31.35)	0.90	18.72 (7.84-27.86)	9.14 (2.72-18.34)	0.11
15	60.48 (29.51-75.16)	28.90 (18.24-96.21)	0.49	75.74 (51.68-134.4)	88.40 (20.15-164.4)	>0.99
30	180.2 (137.9-235.3)	212.1 (142.3-290.8)	0.90	301.8 (182.3-373.4)	264.7 (151.4-446.0)	0.84
45	163.5 (136.6-229.5)	265.4 (185.9-426.2)	0.04	269.0 (190.4-437.2)	296.0 (202.7-476.2)	0.66
60	97.51 (29.14-179.3)	249.3 (172.0-423.3)	0.01	235.3 (118.6- 413.0)	339.0 (199.8-414.9)	0.28
90	53.73 (33.89-111.5)	109.1 (88.87-295.0)	0.02	142.0 (93.42-343.2)	239.3 (131.9-385.8)	0.32
120	7.29 (-7.58-81.84)	51.73 (5.41-113.2)	0.36	102.3 (71.06-308.3)	213.1 (118.0-376.9)	0.11

[1] Data is expressed as median (IQR)

[2] *P*-values are determined with a Mann-Whitney U test

Supplementary Table 5. Differences of number of chews and VAS-scale ratings of satiety, hunger and desire to eat between low and high *AMY*1 CN groups in the meal study[1]

	Low *AMY1* CN (n=9)	High *AMY1* CN (n=10)	*P*-value[3]
40 g starch			
Chews[2]	29.6 (20.0-33.7)	25.5 (21.2-36.9)	0.92
Satiety	27 (11.0-34.2)	36.25 (22.3-46.4)	0.21
Hunger	59.3 (33.6-74.1)	58.6 (48.4-2.7)	0.80
Desire to eat	57.1 (50.1-76.5)	61.5 (50.1-80.0)	0.78
80 g starch			
Chews[2]	27.4 (18.6-30.3)	25.6 (22.8-30.8)	0.88
Satiety	45.1 (32.1-59.1)	47 (36.7-77.8)	0.56
Hunger	48.7 (30.0-3.2)	42.3 (17.3-64.1)	0.84
Desire to eat	53.3 (46.0 -57.0)	41.8 (19.1-62.8)	0.66

[1] Data is expressed as median (IQR)

[2] The average of chews for the first 5 standard bite-size pieces

[3] *P*-values are determined with a Mann-Whitney U test
